# Study on the forming characteristics of polytetrafluoroethylene/copper jet with different preparation processes

**DOI:** 10.1038/s41598-023-43053-6

**Published:** 2023-09-20

**Authors:** Jianya Yi, Ruijie Hao, XueZhi Tang, Siman Guan, Zhijun Wang, Jianping Yin

**Affiliations:** 1 School of Mechatronic Engineering, North University of China, Taiyuan, 030051 People’s Republic of China; 2 Chongqing Hongyu Precision Industry Group Co.Ltd, Chongqing, 402760 People’s Republic of China

**Keywords:** Engineering, Materials science, Physics

## Abstract

In this paper, PTFE/Cu composite material for liner is taken as the research object, and the preparation process and jet forming characteristics of PTFE/Cu composite liner are studied. The liners were prepared by extrusion molding, molded sintering and hot-pressing sintering. Due to different preparation processes, different microstructures of the liner can occur, including defects such as pores and microcracks, resulting in different strength and density of the liner, leading to differences in the forming characteristics of the jet. Therefore, the forming process of the jet was simulated by the finite element numerical simulation software. It was found that there was obvious radial expansion effect in the head of the jet, but with the increase of density, the radial expansion effect was weakened, and the jet velocity decreased gradually. The strength and densification of the shaped charge liner prepared by different processes were different. The densification of the molded sintering liner was generally better than that of the other two kinds of shaped charge liners. As a result, the velocity of the jet formed by the molded sintering liner is always the highest, with a numerical simulation velocity of 6642 m/s and an experimental velocity of 6534.7 m/s. The second is the jet of the hot-pressing sintering liner and the lowest velocity is the jet of the extrusion molding cover, with a numerical simulation velocity of 6482 m/s, while the experimental velocity is only 6397.9 m/s. The jet velocity measured by the pulse X-ray experiment was compared with the velocity of the numerical simulation, and the error was within 2.96%, which verifies the accuracy of the numerical simulation.

## Introduction

The emergence and development of shaped charge effect are more and more widely used in military and civilian fields. As the core part of shaped charge, the liner directly determines the penetration efficiency of shaped charge in many aspects. With the development of material technology and processing technology, in order to obtain good penetration effect, it is not always only the liner made of high-density metal can meet the requirements. For some light protective fortifications and the target of draping reactive armor, the composite material based on PTFE (polytetrafluoroethylene) material has become the preferred choice for the shaped charge liner material. Compared with high-density metal materials, this kind of polymer material has low density, but the jet formed has good stability, high energy and good penetration performance.

At present, many studies have been done on the properties of PTFE-based composites. For example, Ge et al.^1^ studied the impact initiation threshold of polytetrafluoroethylene/aluminum (73.5 wt% and 26.5 wt%) composites by light gas gun impact experiment, and found that the impact initiation of polytetrafluoroethylene/aluminum was determined by both impact pressure and loading strain rate. Chang^2^ modified low-density PTFE by adding a certain proportion of copper powder to the matrix, which improved the density and jet energy of the material and improved the static and dynamic mechanical properties of the material. Xuezhi et al.^3^ prepared two kinds of samples by extrusion molding and hot-pressing sintering, and studied the effect of preparation methods on the compressive properties of Cu-PTFE composites. Rae^4,5^ carried out the tensile experiment and compression experiment of polytetrafluoroethylene samples at different strain rates and temperatures, and used the Hopkinson bar to carry out the compression test of the samples at different strain rates and temperatures. In addition to the mechanical properties test, some scholars have also studied the influence factors of PTFE fracture behavior^6^, preparation process^7^ and filler shape^8^ on the properties. In addition, PTFE-based reactive materials formed by mixing active metal powders in fluoropolymer binders are also a research hotspot, including energy release characteristics^9,10^, microstructure and mechanical properties^11^ and other aspects of these materials.

Xiao et al.^12^ prepared three kinds of reactive liners by pressing and sintering, and studied the damage effect of reactive material shaped charge on multi-layer targets. Guo et al.^13^ studied the influence of the wave shaper on the formation behavior and penetration performance of the reactive liner jet through experiments and simulations, and discussed the wave shaper effect, including the propagation behavior of the detonation wave, the velocity and temperature distribution of the reactive jet, and the penetration depth of the reactive jet. Borkowski et al.^14^ introduced the experimental results of the formation and penetration efficiency of EFP shaped charge liner by powder metallurgy method, and found that the EFP shaped charge liner prepared by powder metallurgy technology can completely replace the traditional spherical liner. Yi et al.^15^ selected three kinds of polymer materials with different properties, polytetrafluoroethylene (PTFE), nylon (PA) and polycarbonate (PC), as the liner materials. Through the combination of numerical simulation and experiment, it was found that the polymer jets of different materials all showed a certain degree of expansion, but due to the difference of material properties, the expansion diameter and head densification degree of the jet were different.

The jet formed by the PTFE composite material as the liner has better performance than the traditional metal jet in some aspects when penetrating the target. The jet formed by the Al/PTFE reactive material liner prepared by cold pressing sintering has a larger aperture and a lower penetration depth for the penetration of thick steel plates compared with the traditional metal liner shaped charge jet^16^. Compared with the traditional metal liner, the reactive material liner has a stronger penetration aftereffect, so it is gradually used as the liner material^17^. Yi et al.^18^ studied the jet formed by PTFE liner and pure copper liner, and found that the polymer expansion jet has a larg-er penetration aperture than the typical copper jet penetration performance. Hirsch and Sadwin^19^ pointed out that a cavity with increased diameter will be formed near the bottom of the penetration hole of the jet formed by the shaped charge liner made of highly compressible materials (such as thermoplastics), which will lead to the failure of brittle materials such as concrete.

The research on the jet forming process generally includes three methods: theoretical analysis, numerical simulation and experimental verification. The use of these three methods is generally based on theoretical analysis, followed by numerical simulation analysis. Finally, the experiment is carried out, and the numerical simulation is adjusted and corrected according to the experiment results. At present, the theory of jet forming process is mainly PER theory, which is more realistic to the reaction of jet forming process. The numerical simulation of jet forming is mainly based on Autodyn or LS-DYNA numerical simulation software. Finally, the actual forming process of jet is verified by experiments.

In this paper, the numerical simulation of jet forming of different PTFE/Cu material liners was carried out based on AUTODYN software. The effects of different liner preparation processes on jet forming characteristics were compared and analyzed. Then, the pulse X-ray experiment was carried out, and the results of numerical simulation analysis were verified according to the experiment results. Finally, according to the numerical simulation and experiment results, the forming principles and characteristics of PTFE/Cu material jets with different preparation processes were summarized.

## Material

For PTFE powder itself, it is insoluble in any solvent and is not easy to be corroded by other substances. Its surface tension is the smallest among solid materials, and it does not adhere to any substance. It has excellent chemical stability and good mechanical toughness at low temperature.

At present, there are many modification methods for PTFE: surface modification, blending modification and filling modification. Because the surface activity of PTFE is very inactive and its viscosity is also very poor, it is very difficult to compound with other materials. That is to say, the biggest technical difficulty of PTFE surface modification is the bonding between materials. In response to this difficulty, the current proposed solution is to increase the surface tension of PTFE as much as possible, and then use an appropriate amount of adhesive to bond it. Blending modification is generally based on the unique material properties of PTFE to alloy some resin and polymer materials, so that the friction and wear properties are optimized, and the toughness and creep resistance are improved. Filling modification refers to the filling technology of filling metal and inorganic materials into PTFE, or filling PTFE with composite materials. Based on the inherent physical and chemical properties of PTFE, when filling it, the filler needs to meet the following requirements: It does not react with PTFE; The performance is stable at 350–380 °C, and there is no decomposition, combustion or explosion reaction. In the process of processing and preparation, it has a high matching degree with PTFE particle size and will not produce cluster phenomenon. It is not easy to get wet.

Therefore, in this paper, PTFE/Cu composites with different mass ratios were prepared by extrusion molding, molded sintering and hot-pressing sintering, and the sintering temperature was not more than 380 °C.

### Extrusion molding

Extrusion molding method refers to a kind of processing and preparation method that plastic materials form products from the hole mold under the strong external force. It mainly includes four steps: (1) Heating and melting the polymer; (2) Conveying or pumping the molten polymer to the forming unit; (3) Molding the melt to achieve the desired shape and size; (4) Cooling and curing.

**Figure 1:**
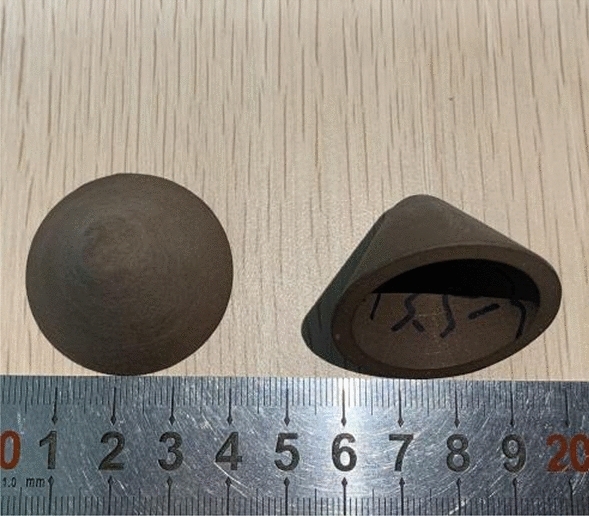
Extrusion molding liner.

Considering the great difference in material properties between PTFE and Cu, PTFE/Cu rods with different densities were directly processed by twin-screw extruder, and then the rods were turned to process the PTFE/Cu liner required for the experiment, as shown in Fig. 1.

### Molded sintering

The preparation process of the molded sintering liner must first put the predetermined mixed powder into the mold with a certain shape, and obtain the product by applying external force to it. After the preform is completed, the subsequent high temperature sintering is needed. This step is the last and most critical step in the whole preparation process. In this step, the material is heated to strengthen the mutual connection between the powder particles so as to achieve the required mechanical properties.

**Figure 2:**
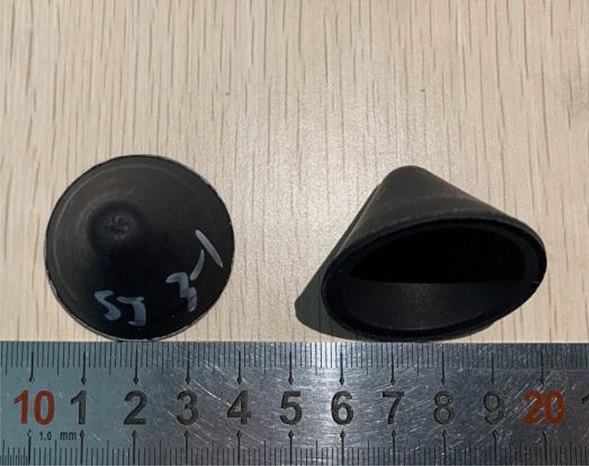
Molded sintering liner.

PTFE/Cu composites were sintered by high temperature sintering furnace. The device was mainly composed of sintering furnace chamber and control panel. During sintering, the molded preform is placed in the furnace cavity, and then the sintering temperature is determined to be 380 °C according to the melting point of PTFE. The temperature change during the sintering process is programmed by the control panel. The heating rate is 60 °C/h. When the temperature reaches 380 °C, the holding stage is entered for 2 h, and then the cooling rate is 60 °C/h. When the temperature is reduced to 310 °C, the holding stage is entered for 2 h, and finally the furnace is cooled to room temperature. Figure 2 is the PTFE/Cu shaped charge liner obtained by molded sintering.

### Hot-pressing sintering

Hot pressing sintering refers to the process of filling the dry mixed powder into the model and heating it through heat conduction or heat radiation. At the same time, the mold is subjected to unidirectional or bidirectional pressure, so that the molding and sintering are completed simultaneously. Due to the simultaneous application of temperature and pressure, the contact, diffusion and flow characteristics of the powder particles are improved, which makes the powder in a thermoplastic state, the deformation resistance is small, and it is easy to plastic flow and densification.

**Figure 3:**
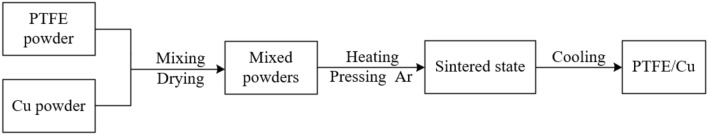
Preparation process of hot-pressing sintering method.

**Figure 4:**
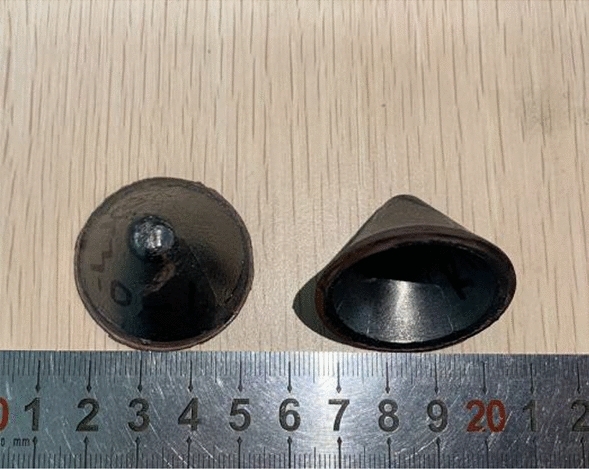
Hot-pressing sintering liner.

Figure 3 shows the process of preparing PTFE/Cu composites by hot-pressing sintering. In order to ensure that the temperature change during the experiment is stable, the whole preparation process is carried out in an argon atmosphere, and the pressure is constant. Figure 4 shows the liner prepared by hot-pressing sintering.

## Numerical simulation of PTFE/Cu jet forming

In order to better analyze the characteristics of jet forming of PTFE/Cu materials with different preparation processes, the numerical simulations of different PTFE/Cu material liners were carried out, and the jet forming processes within 60 μs were compared and analyzed. Considering the feasibility of different preparation processes for the manufacture of liners, only two kinds of density can be prepared by extrusion, which are 3.0 g/cm^3^ and 3.5 g/cm^3^, respectively. There are three kinds of density for molded sintering and hot-pressing sintering, which are 3.0 g/cm^3^, 3.5 g/cm^3^ and 4 g/cm^3^, respectively.

**Figure 5:**
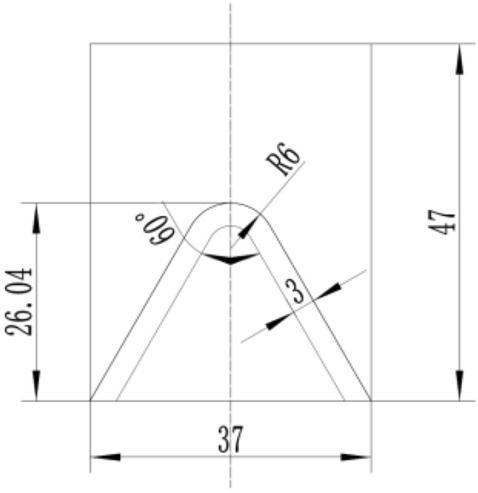
Structure diagram of shaped charge.

In this section, the numerical simulation of PTFE/Cu liner jet forming was carried out by SPH method. The structure of shaped charge is a conical liner without shell and equal wall thickness, in which the cone top is rounded, as shown in Fig. 5. Among them, the wall thickness of the liner is δ = 3 mm, the cone angle of the liner is 2α = 60°, the height of the liner is l = 26.04 mm, the diameter of the charge is D_k_ = 37 mm, and the height of the charge is H = 47 mm.

**Figure 6:**
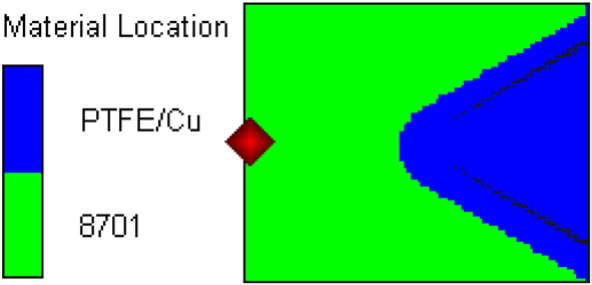
Finite element model.

The SPH algorithm in AUTODYN software is used to simulate the jet forming process. There is no boundary problem. The gap of SPH particles is 0.05 cm, and the mesh size of the target plate is 0.05 cm. Due to the symmetry of the whole model, in order to improve the calculation efficiency and make the jet forming process more intuitive, a 1/2 three-dimensional finite element model is established, as shown in Fig. 6.

The constitutive model of PTFE/Cu material is Johnson–Cook, and the state equation is Shock. In this paper, the constitutive equations of all PTFE/Cu materials are fitted by ourselves. The dynamic and static mechanical properties of the material samples are tested at room temperature. The constitutive equations of PTFE/Cu materials at room temperature are fitted without considering the influence of temperature softening effect. The specific parameters are shown in Table 1.

**Table 1:** Shock equation of state parameters and Johnson–Cook parameters of PTFE/Cu material.

Liner No	Preparation process	$\rho$ (g/cm^3^)	Gruneisen coefficient	*C*_1_	*S*_1_	Yield strength (MPa)	Hardening constant (MPa)
1#	Extrusion molding	3	2.7482	2.1175	1.7329	14.35	16.306
2#		3.5	2.7915	2.1792	1.1618	9.73	41.897
3#	Molded sintering	3	2.7482	2.1662	1.7472	12.16	40.083
4#		3.5	2.7915	2.1218	1.7064	16.72	48.122
5#		4	2.8073	2.0732	1.8137	20.396	60.039
6#	Hot-pressing sintering	3	2.7482	2.1433	1.7045	17.13	32.726
7#		3.5	2.7915	1.9671	2.2286	20.31	34.507
8#		4	2.8073	2.0327	1.7881	19.47	46.680

Explosive 8701 is selected as the explosive, and JWL state equation is used to describe the detonation process of explosive. The relevant material parameters of explosive are derived from the material library of AUTODYN software. The specific parameters are shown in Table 2.

**Table 2:** JWL equation of state parameters and C–J parameters of 8701 explosive.

*A *(GPa)	*B *(GPa)	*R*_1_	*R*_2_	*W*	*ρ *(g/cm^3^)	*D *(m/s)	*E *(KJ/m^3^)	*P*_*CJ*_ (GPa)
854.5	20.493	4.6	1.35	0.25	1.71	8315	8.5e6	29.5

In the table: *ρ* is the average density of the main charge prepared for the experiment, *D* is the detonation velocity, *E* is the energy density per unit volume, and *P*_*CJ*_ is the detonation pressure of the explosive.

## Pulse X-ray experiment

Pulse X-ray photography is also a kind of high-speed photography. It generates X-ray pulses with extremely short time through high-voltage pulse generators and pulse X-ray tubes. The pulse X-ray is different from the general high-speed photography, mainly because the pulse width of the X-ray source is very narrow, which can penetrate the smoke and dust generated by explosion and high-speed impact, and record the shape or trajectory of the jet or fragment. The forming principle of pulse X-ray is that when the X-ray beam projects an object, high-energy and short-wavelength rays can penetrate the object to a certain extent. The amount of X-ray through the object depends on the energy of the radiation and the density and thickness of the object. Low-energy radiation is more easily absorbed than high-energy radiation, and objects with high density or thickness are more easily absorbed. The emitted X-ray beam is modulated so that different object shapes can be displayed on the film.

In this paper, the pulse X-ray experiment uses HP43737 type 300 kV pulse X-ray, the output current is 5 KA, and the pulse width is 50 ns. In each experiment, the pulse X-ray is triggered twice at intervals and imaged on the same film. The shape and appearance of the jet and the forming process can be clearly seen on the image. According to the distance between the jet head positions in the two images, the actual length of the jet head is calculated. Then, according to the time interval between the two triggers, the average velocity of the jet in this time period can be calculated.

**Figure 7:**
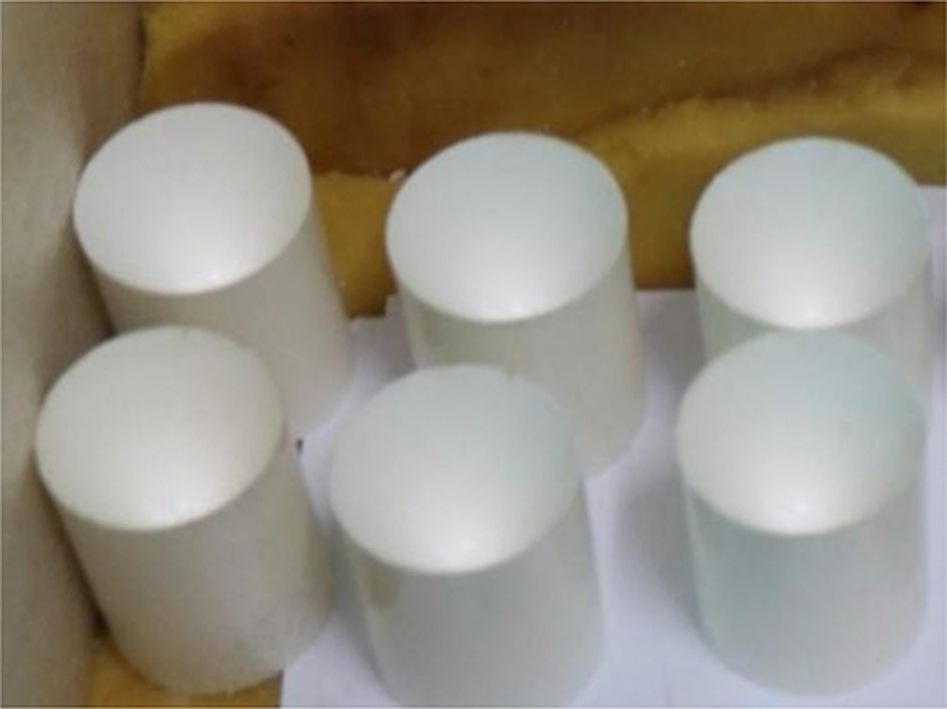
Explosive of jet forming experiment.

**Figure 8:**
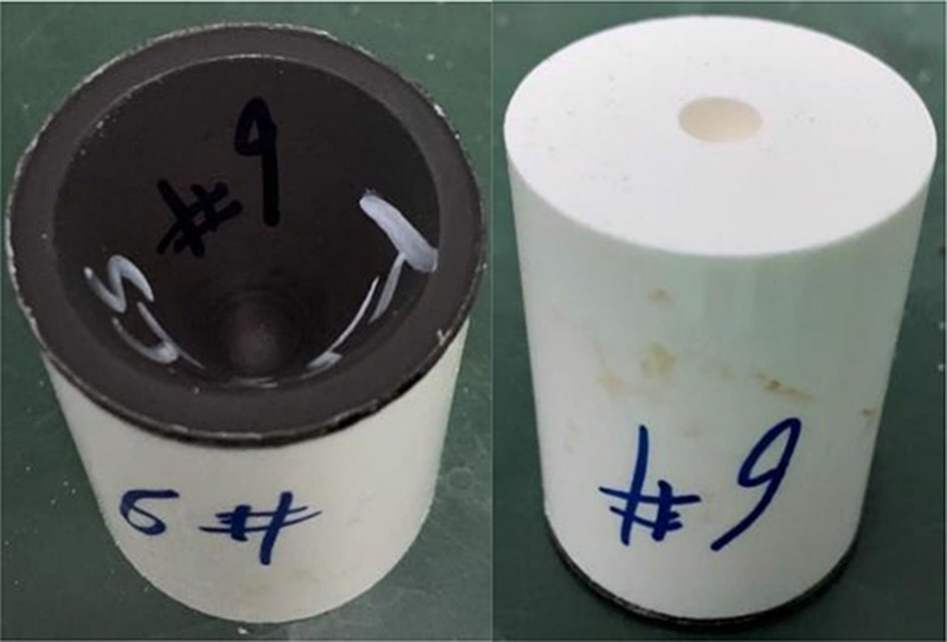
Shaped charge structure.

According to the preparation process described above, the PTFE/Cu shaped charge liners required for the experiment were prepared respectively. The cylindrical 8701 explosive required for the experiment was pressed out by using the specified pressing mold, as shown in Fig. 7. The explosives used in the experiment were respectively detonated at the center point. The liner and explosive were bonded by a certain ratio of shellac paint. The bonded shaped charge structure is shown in Fig. 8.

**Figure 9:**
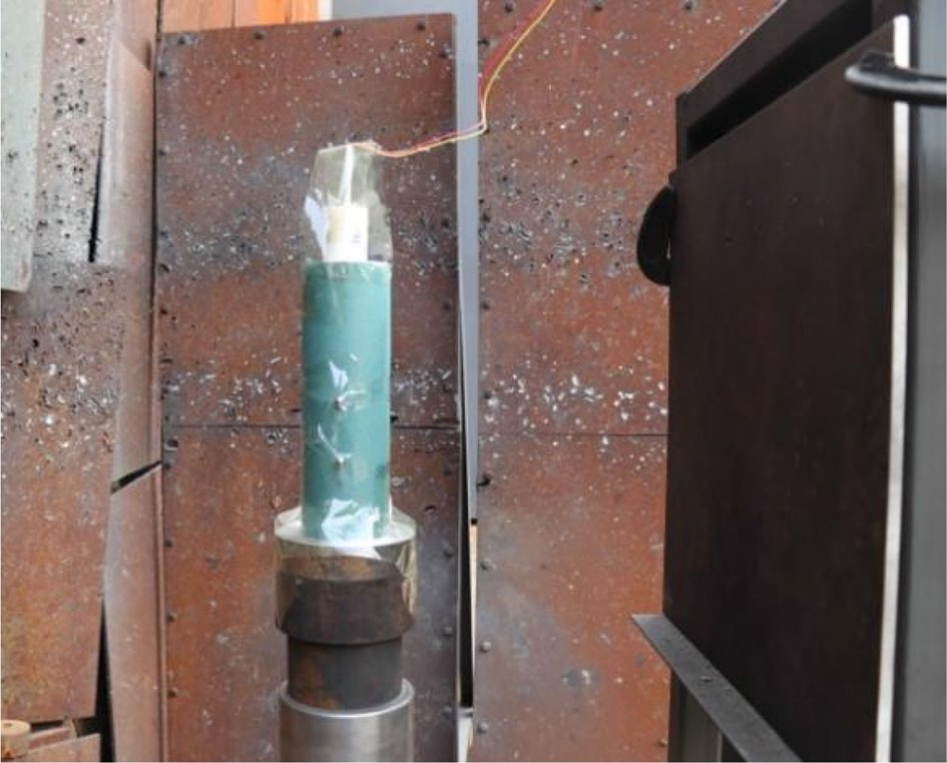
Arrangement of charge and stand-off barrel.

**Figure 10:**
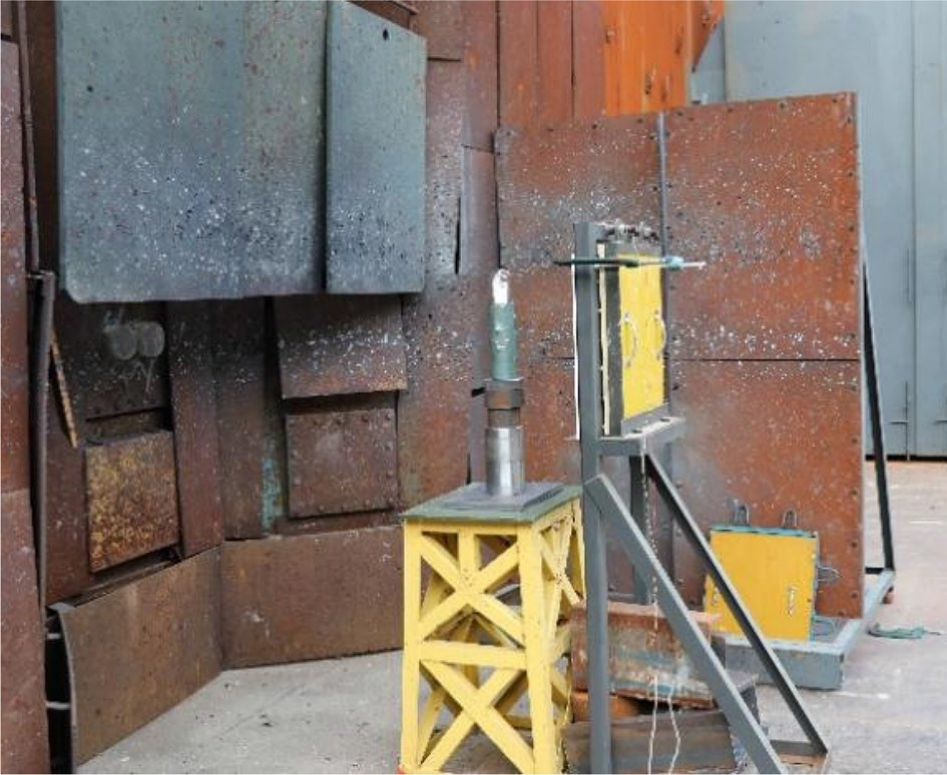
Setting of pulse X-ray experiment.

In order to make the pulse X-ray can fully capture the forming process of the jet, the distance between the top of the liner and the aftereffect target was set to 6 times the charge diameter, that is, the stand-off was 222 mm. According to the forming principle of pulse X-ray, in order to obtain a clear image as much as possible, it is necessary to make the density of the stand-off barrel lower than the jet density. Because the minimum density of the liner is 3 g/cm^3^, in order to form a certain density difference, the material of the stand-off barrel in this experiment is barley paper, and its density is 1.1 g/cm^3^. Before the experiment, two steel balls need to be bound on the top of the stand-off barrel, as shown in Fig. 9. The function is to be used for distance positioning during imaging, so as to determine the proportional relationship between the length on the negative and the actual length. The distance between the two steel balls is 50 mm. The specific layout of the pulse X-ray experiment site is shown in Fig. 10.

In addition, it is also necessary to determine the trigger time of the two shots. If the trigger time is too late, the jet will hit the steel target, and the captured image is not pure jet forming. If the trigger time is too early, the liner has not been completely crushed, then the calculated speed is not the speed after the jet forming is stable. Combined with the simulation results and considering the time of the experiment, the two trigger times that can be selected for the pulse X-ray are determined, which are 10 μs, 15 μs or 30 μs after detonation. The density of the liner in this X-ray experiment is only 3.5 g/cm^3^ and 4 g/cm^3^. The specific experiment plan is shown in Table 3.

**Table 3:** Experiment parameter setting.

Liner no.	Preparation process of liners	Liner density (g/cm^3^)	Trigger time 1 (μs)	Trigger time 2 (μs)
2#	Extrusion molding	3.5	15	30
4#	Molded sintering	3.5	10	30
5#	Molded sintering	4	15	30
7#	Hot-pressing sintering	3.5	15	30
8#	Hot-pressing sintering	4	15	30

## Results and discussion

### Numerical simulation results

#### Effect of density on PTFE/Cu jet forming characteristics

In order to study the influence of density change on jet forming in the preparation process of the same liner, the expansion of detonation products and the forming state of jet at different times were observed. According to the jet forming process, the velocity contours of 5 μs, 10 μs, 20 μs, 30 μs, 40 μs and 50 μs were recorded respectively, and then compared and analyzed.

**Figure 11:**
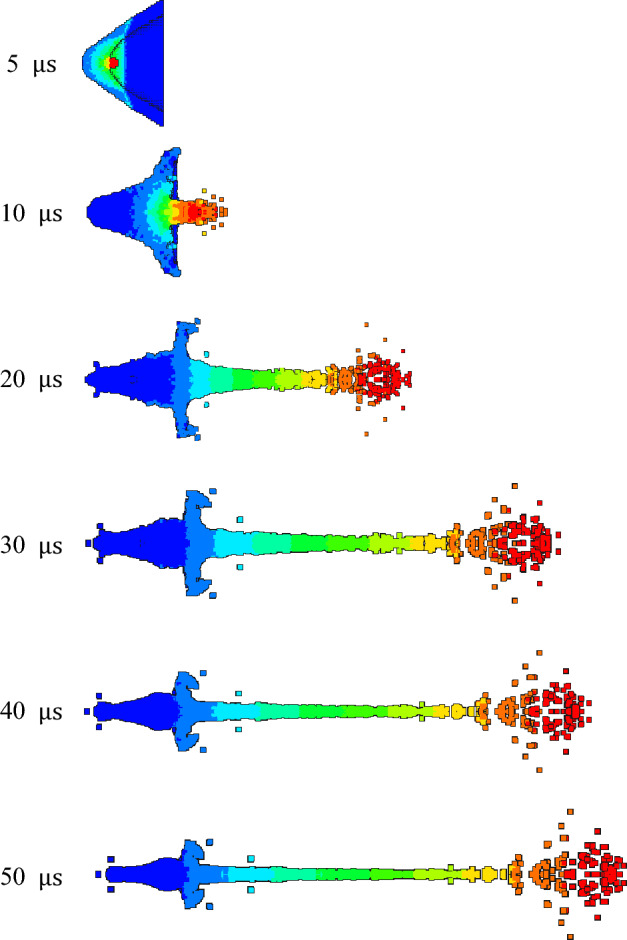
Jet forming process of 1#-PTFE/Cu liner (extrusion molding, $\rho$ = 3 g/cm^3^).

**Figure 12:**
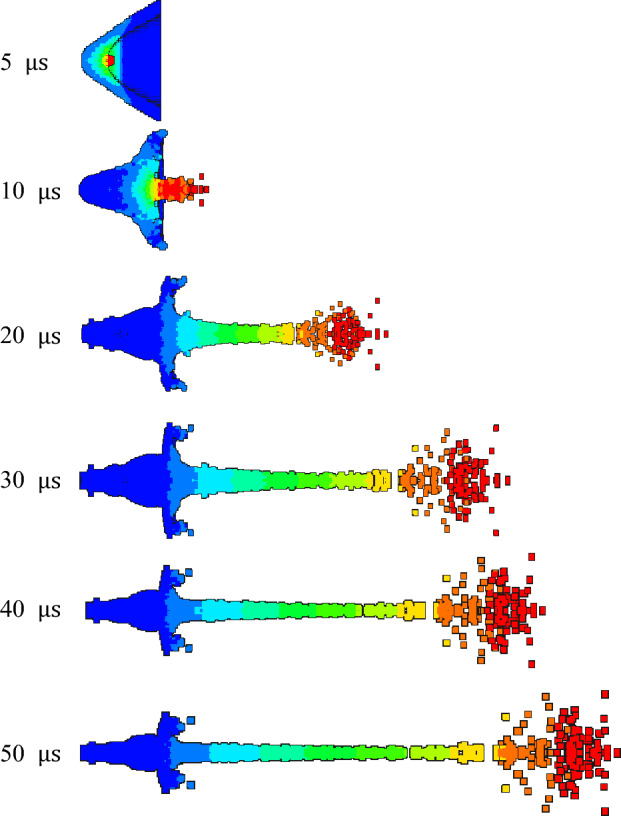
Jet forming process of 2#-PTFE/Cu liner (extrusion molding, $\rho$ = 3.5 g/cm^3^).

As shown in Figs. 11 and 12, the velocity gradient nephograms of the jet forming process of 1# liner and 2# liner are respectively shown. Due to the gradient of velocity, the color of the jet varies. Each color from the jet head to the slug represents a velocity gradient, which is the first and second velocity gradients, representing the gradient change in velocity from high to low. The density of 1# liner is 3 g/cm^3^, and the density of 2# liner is 3.5 g/cm^3^. It can be seen from the figure that the formation of the jet mainly includes four processes: explosive detonation, liner collapse, initial formation of the jet and continuous stretching of the jet. It can be seen from Fig. 11 that at 5 μs, the shaped charge explosive is completely detonated, and the liner is gradually crushed under the action of detonation wave. At 10 μs, the liner is completely crushed, and then the liner gradually converges along the axis to form a jet and slug. At 20 μs, the shape of the jet is basically formed. With the passage of time, the jet forming gradually stabilizes, and due to the existence of velocity gradient, the jet head appears to be broken in the later stage of forming. Comparing Figs. 11 and 12, it can be seen that the shape of the jet begins to differ at 20μs, that is, the effect of density on the shape of the jet starts from its formation. When the liner density is 3 g/cm^3^, the head expansion phenomenon of the jet is more obvious than that when the density is 3.5 g/cm^3^. This is because the increase of density will lead to the decrease of PTFE content and the increase of Cu content, and the presence of polymer PTFE will cause the jet to produce head expansion. The jet head of the liner with a density of 3.5 g/cm^3^ has better cohesiveness. This is because the Cu content in the liner material is close to the PTFE content. Cu has good plasticity and can form a jet with good adhesion. With the increase of density and copper content, the formed jet can reflect the characteristics of copper jet more and more. In addition, the jet formed by the two liners began to be broken at 30 μs, and at 50 μs, the jet appeared multiple necking and breaking.

Table 4 shows the characteristic parameters of the jet forming stability. It can be seen that at the same time point, with the density increasing, the jet head velocity decreases, the jet length decreases, and the jet head diameter becomes smaller. In addition to the density, the characteristics of the raw material itself also affect the results.

**Table 4:** Jet forming characteristic parameters of extrusion molding liner with different density (t = 30 μs).

Liner density (g/cm^3^)	3	3.5
Jet head velocity (m/s)	6752	6482
Jet length (mm)	164.28	158.78
Jet head diameter (mm)	28.02	25.78

**Figure 13:**
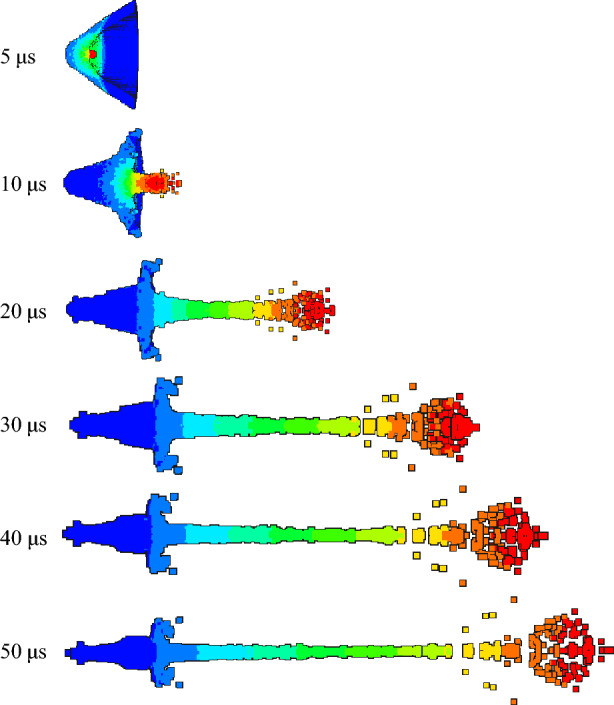
Jet forming process of 6#-PTFE/Cu liner (molded sintering, $\rho$ = 3 g/cm^3^).

**Figure 14:**
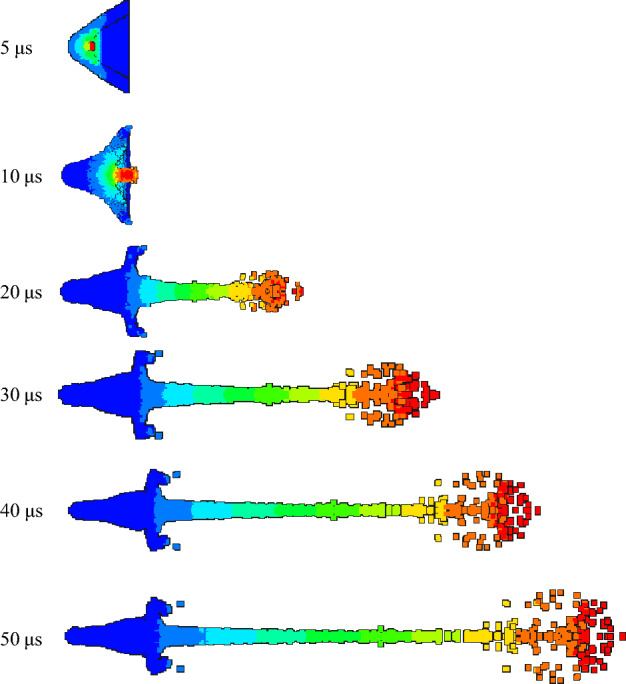
Jet forming process of 7#-PTFE/Cu liner (molded sintering, $\rho$ = 3.5 g/cm^3^).

**Figure 15:**
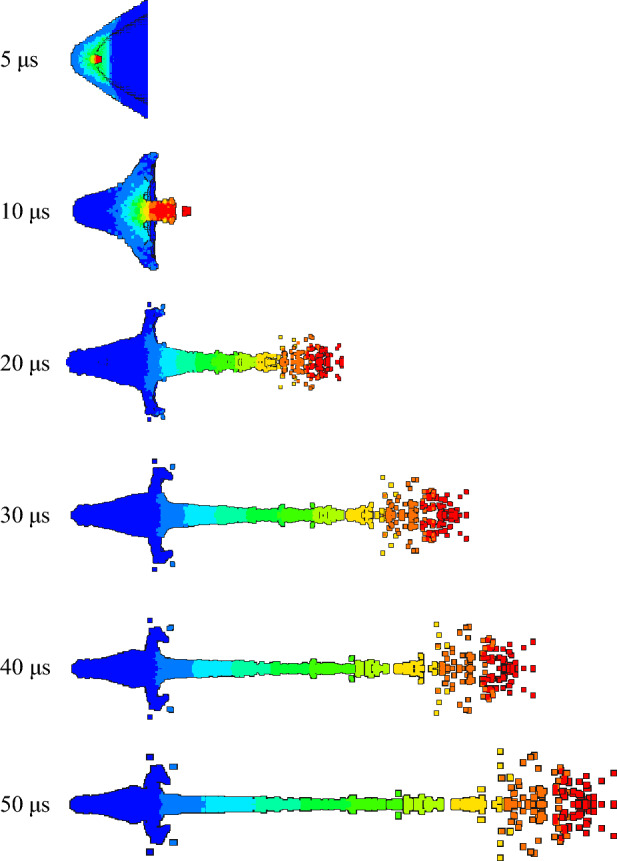
Jet forming process of 8#-PTFE/Cu liner (molded sintering, $\rho$ = 4 g/cm^3^).

The jet forming characteristics of the molded sintering liner at different time were analyzed, as shown in Figs. 13, 14 and 15.

From the above three figures, it can be seen that for the molded sintering liner, the forming process of the liner is consistent with that of extrusion forming liner, which is divided into four parts. With the increase of density, there are obvious differences in the morphology of the jet of the molded sintering liner with different densities. It can be seen from the diagram that the jet formed by the 6# liner is stable at 20 μs and the jet head has obvious radial expansion. With the passage of time, the radial expansion of the head gradually becomes larger, and the jet appears obvious necking at the critical point of different velocity gradients. At 40 μs, the jet appears obvious fracture, the fracture position is the velocity gradient node, and the jet head is obviously not condensed. At 50 μs, the jet appears multiple fractures, except for the velocity gradient node. The jet fracture in the second and third velocity gradients is also very serious, and the “bone-shaped” fracture jet appears. In addition, the jet head expands, the whole is discrete, and the particle sense is obvious. For the 7# liner, it can be seen from Fig. 14 that the radial expansion of the jet is not obvious at 20 μs. As time goes on, the jet gradually stretches, and there are many obvious necking phenomena at 40 μs. At the same time, the jet accumulates at the head. At 50 μs, the jet breaks and the head accumulates obviously. This is because in the liner with this density, the PTFE content is close to the Cu content, and the jet characteristics formed are between pure PTFE and Cu. Therefore, the jet accumulates at the head, but the continuity of the jet is higher than that of the 6# liner. The expansion of the jet head of the 8# liner is weakened, and there is no accumulation, and the jet fracture phenomenon is obvious.

Table 5 is the characteristic parameters of the jet at 30 μs for the molded sintering liner. It can be seen from the table that with the increase of Cu content, the peak velocity of the jet head decreases, while the Cu content increases, and the jet length gradually decreases. When the density is 3 g/cm^3^, the maximum jet length is 162.91 mm. According to the graphic conclusion, as the density increases, the radial expansion of the jet head gradually weakens, so the head diameter of the jet also gradually decreases.

**Table 5:** Jet forming characteristic parameters of molded sintering liner with different density (t = 30 μs).

Liner density (g/cm^3^)	3	3.5	4
Jet head velocity (m/s)	6917	6642	6380
Jet length (mm)	162.91	157.65	150.45
Jet head diameter (mm)	25.14	23.14	20.60

**Figure 16:**
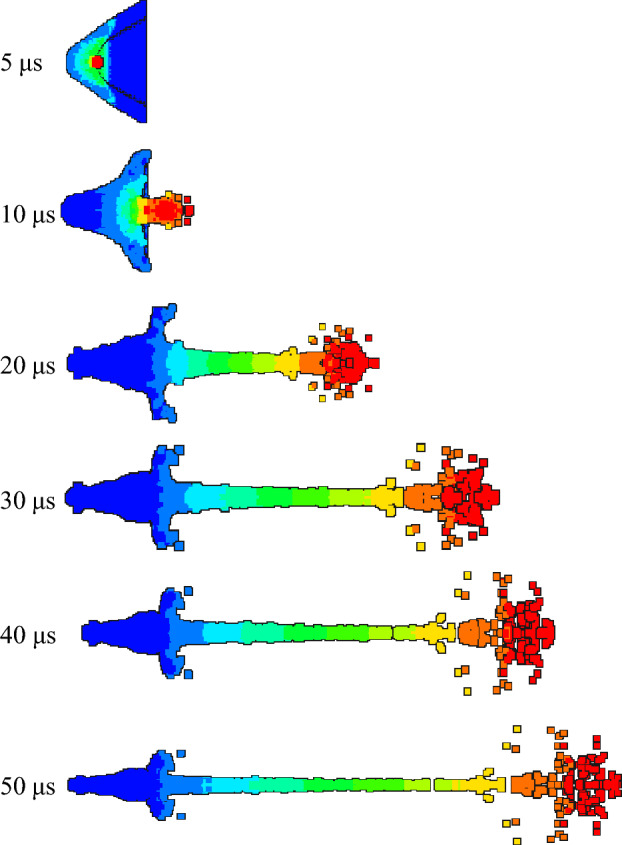
Jet forming process of 9#-PTFE/Cu liner (hot-pressing sintering, $\rho$ = 3 g/cm^3^).

**Figure 17:**
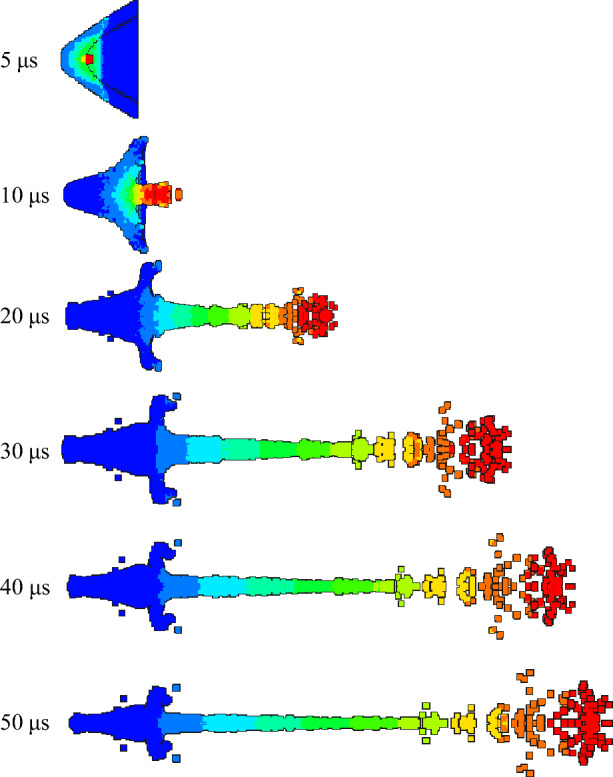
Jet forming process of 10#-PTFE/Cu liner (hot-pressing sintering, $\rho$ = 3.5 g/cm^3^).

**Figure 18:**
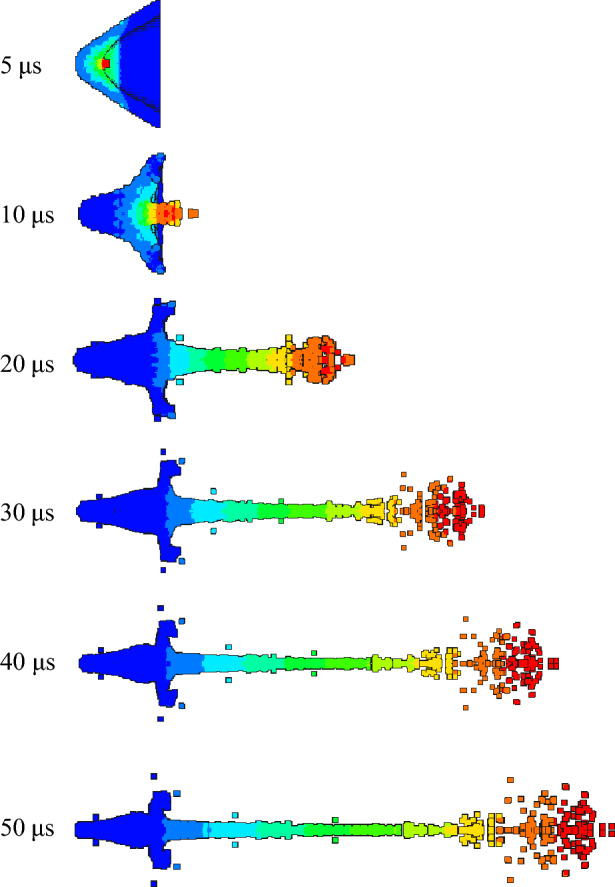
Jet forming process of 11#-PTFE/Cu liner (hot-pressing sintering, $\rho$= 4 g/cm^3^).

Figures 16, 17 and 18 are the morphological characteristics of the hot-pressing sintered liner during the jet forming process, respectively.

As shown in the above figure, the jet forming process of the hot-pressing sintering liner is consistent with the jet forming process of the extrusion molding and the molded sintering liner. As the density increases, the jet formed by the hot-pressing sintering liner gradually becomes continuous. From Fig. 16, it can be seen that when the density is 3 g/cm^3^, the accumulation of the jet head is obvious, but it gradually dissipates with the passage of time. At 30 μs, the jet begins to have a necking trend. At 40 μs, the necking phenomenon of the jet is obvious, which mainly occurs in the second and third velocity gradients. When the time reaches 50 μs, the jet is obviously broken, and the first, second and third velocity gradients all appear “bone” type fracture. This phenomenon is obviously different from the jet characteristics formed by the same density liner of the first two preparation processes. It can be seen from Fig. 17 that when the density is 3.5 g/cm^3^, with the increase of time, the jet head accumulates, the divergence is obvious, and the fracture is obvious. At 20 μs, the jet begins to neck. At 30 μs, the first and second velocity gradients of the jet break, and the head diverges obviously. At 50 μs, the jet head breaks obviously, with slight radial expansion, and the nodes inside the second velocity gradient, the second and third velocity gradients break. The necking of the third velocity gradient is serious. With the increase of time, the subsequent jet break may occur. However, in general, the continuity of the jet is worse than that of the jet formed when the liner density is 3 g/cm^3^. When the density is 4 g/cm^3^, the continuity of the jet is the best, and the radial expansion of the jet head is relatively less obvious during the forming process. At the same time, the occurrence time of the jet necking is later than that of the jet of the previous two densities, and there is no breaking. The appearance of the forming is closer to the appearance of the ordinary metal jet.

Table 6 shows the forming characteristic parameters of hot-pressing sintering liner jet under different densities. It can be seen that as the density increases, the head velocity of the jet decreases, and the jet length also decreases with the increase of the density. When the density of the liner is 3 g/cm^3^, the length of the jet reaches a maximum of 158.617 mm, and the diameter of the jet head gradually decreases. This also shows that as the density increases, the jet formability is closer to the metal jet.

**Table 6:** Jet forming characteristic parameters of hot-pressing sintering liner with different density (t = 30 μs).

Liner density (g/cm^3^)	3	3.5	4
Jet head velocity (m/s)	6769	6507	6236
Jet length (mm)	158.617	151.87	146.62
Jet head diameter (mm)	25.24	21.03	20.42

### Effect of preparation process on PTFE/Cu jet forming characteristics

In order to study the influence of different preparation processes of liner on jet forming characteristics, the jet formed by liners processed by different preparation processes under the same density was analyzed, and the change of head velocity and jet length in the process of jet forming were studied.

**Figure 19:**
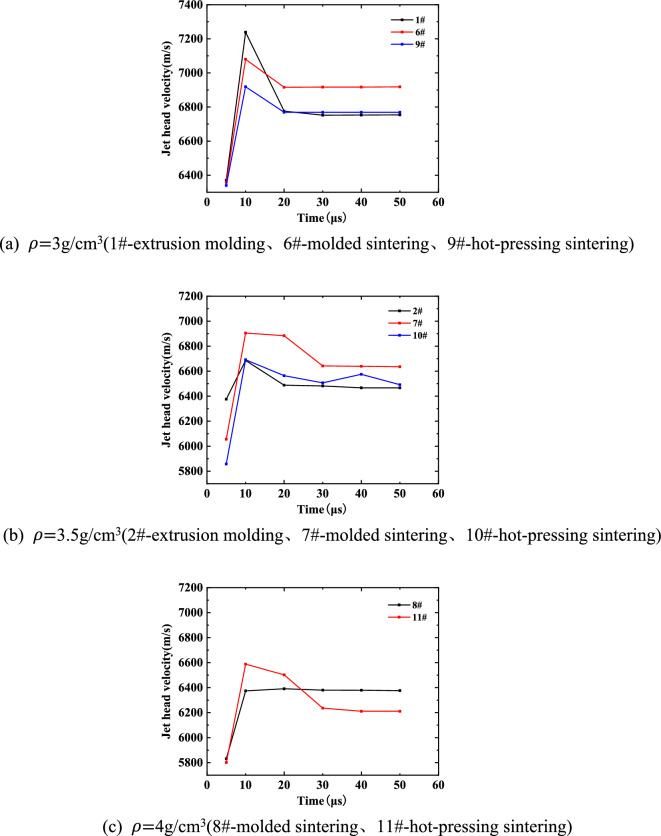
Jet velocity under different preparation processes.

As shown in Fig. 19, the change of the head velocity of the same density liner in the jet forming process under different preparation processes is shown. It can be seen from the diagram that when the density of the liner is 3 g/cm^3^, the jet forming speed of the molded sintering liner is higher than that of the extrusion molding liner and the hot-pressing sintering liner. At 5 μs, the explosive is in the initial state of complete detonation. At this time, the velocity of the jet formed by the three kinds of liners with a density of 3 g/cm^3^ is not much different, which is close to 6300 m/s. However, when it reaches 10 μs, the velocity of the jet head increases, and the velocity of the jet head of the molded sintering and hot-pressing sintering liners is significantly lower than that of the extrusion molding. This is because the interface bonding strength of the particles is improved by pressing and then sintering, and a new material is generated to improve the strength and plasticity of the material. The thermal mismatch between the sintered PTFE and Cu leads to many dislocations, which indirectly enhances the properties of the composites^20^. Therefore, when the density of the liner is 3 g/cm^3^, the content of Cu is lower than that of PTFE, and there are fewer new materials formed during the molded sintering process. The strength of the liner is lower than that of hot-pressing sintering, but higher than that of extrusion molding liner. In the process of the liner crushing to the gradual formation of the jet (10 μs), the lower the strength, the higher the head speed. Only holes are observed in the microstructure of the molded sintering liner, while in addition to holes, there are obvious circumferential cracks in the microstructure of the hot-pressing sintering liner. Therefore, it is considered that the densification of the molded sintering liner is better than that of the hot-pressing sintering, and the densification of the hot-pressing sintering liner is better than that of the extrusion molding liner. In the tensile process of the jet, the higher the densification, the less obvious the head accumulation phenomenon, and it can be considered that the head mass is smaller, so the head speed is higher. Therefore, from 20 μs backward, the jet velocity of extrusion molding and hot-pressing sintering liner is close to the same, which is lower than that of molded sintering jet.

When the density of the liner is 3.5 g/cm^3^, the change speed of the jet head of the different process liners is different from the value when the density is 3 g/cm^3^. When the time is 5 μs, the explosive detonation, the jet begins to form. The jet head speed of the extrusion molding liner is the largest, and the jet head speed of the hot-pressed sintering liner is the smallest. When the time is 10 μs, the head speed of the three jets shows an upward trend. With the passage of time, the jet head speed of the molded sintered liner has always been the largest, while the jet forming speed of the extrusion molding and hot-pressed sintering has little change, basically the same. When the density is 3.5 g/cm^3^, the content of PTFE is the same as that of Cu, and the new material formed in the process of molding sintering increases. The strength and densification of the liner are higher than those of extrusion molding and hot-pressing sintering. Therefore, after the jet is gradually formed, the head velocity of the jet formed by molding is always higher than that of extrusion molding and hot-pressing sintering.

When the density of the liner is 4 g/cm^3^, due to the different molding difficulties of the liners prepared by different preparation processes, it is impossible to process the extrusion molding liner under the density. Therefore, only the jet head velocity of the two liners of the molded sintering and hot-pressing sintering is compared. It can be seen that the initial jet head velocity is basically the same, both of which are about 5800 m/s. With the passage of time, the jet head velocity of the hot-pressing sintering liner begins to be higher than the jet head velocity of the molded sintering liner. After about 25 μs, it is lower than the jet head velocity of the molded sintering liner. This is different from the change of jet head velocity in the other two densities. Because the content of Cu is higher than that of PTFE when the density of the liner is 4 g/cm^3^, the jet characteristics are closer to the metal jet, and the density of the liner is better, but the strength is not as good as hot-pressing sintering. Once the jet begins to stretch, the jet head velocity of the molded sintering liner is lower than that of the hot-pressing sintering jet, and there is no obvious accumulation phenomenon in the head, so the velocity changes little. However, the densification of the hot-pressing sintering liner is poor, and the head velocity is higher in the initial stage. With the stretching of the jet, the jet head gradually accumulates and the velocity begins to decrease.

In addition, with the increase of density, the peak velocity of the jet head of the same type of liner decreases gradually. This is because with the increase of density, the Cu content of the liner increases gradually, the forming dispersion of the jet is weakened, the cohesion is enhanced, the density of the jet head increases, and the detonation velocity of the explosive is constant, so the velocity decreases. In addition, although the sound velocity of Cu is higher, the increase of Cu content in the liner does not make the sound velocity of PTFE/Cu material greatly improved, so the size of the jet velocity is mainly determined by the density of the liner.

**Figure 20:**
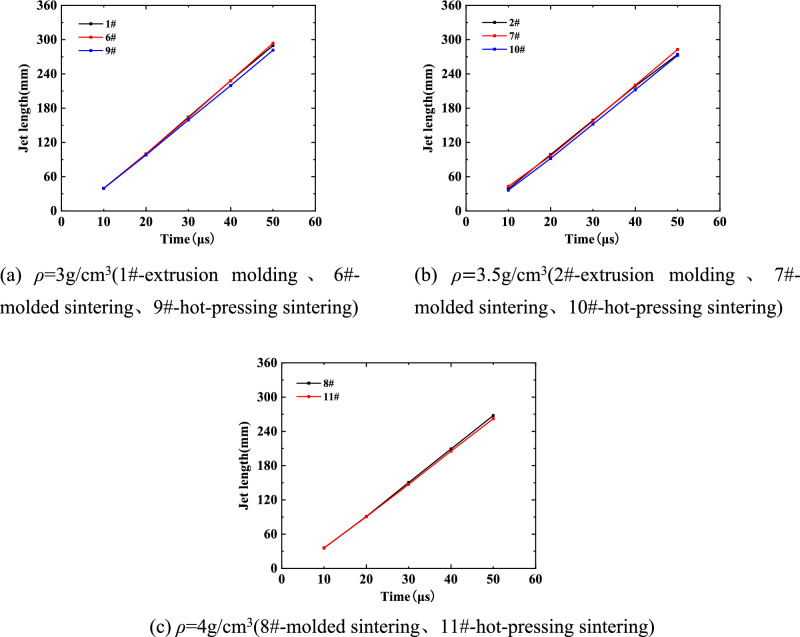
Variation of jet forming length of liner with different preparation processes.

As shown in Fig. 20, the jet length changes of the same density liner at different time points under different preparation processes. Among them, 1#, 6# and 9# are extrusion molding, molded sintering and hot-pressing sintering liners with a density of 3 g/cm^3^, respectively; 2#, 7# and 10# are extrusion molding, molded sintering and hot-pressing sintering liners with a density of 3.5 g/cm^3^, respectively; 8# and 11# are molded sintering and hot-pressing sintering liners with a density of 4 g/cm^3^, respectively. It can be seen from the diagram that when the density of the liner is 3 g/cm^3^, the length of the molded sintering jet in the molding process is the largest, followed by extrusion molding, and finally hot-pressing sintering. In other words, in addition to the density, the length of the jet is also affected by the process. When the time is 10 μs, the jet length values of the three liners are almost the same, all of which are about 40 mm. This is because the material properties of the liner are different, and it is in the early stage of jet forming, mainly depending on the speed brought by the detonation of the explosive to the liner. With the passage of time, the length of the jet gradually increases. At 50 μs, the lengths of the three jets are 289.52 mm, 293.72 mm and 281.48 mm, respectively. When the density is 3.5 g/cm^3^, the length of the initial stage of jet forming is relatively close. With the increase of time, the difference in length gradually appears. Among them, the jet length of the extrusion molding liner is greater than the other two jets. At 50 μs, the lengths of the three jets are 274.1 mm, 282.74 m and 272.36 mm, respectively. Because the mass ratio of PTFE and Cu materials in the density formula is close, the divergence of the jet head is not obvious and the cohesion is enhanced. Considering the influence of different processes, the advantage of jet length formed by molded sintering liner can show that the liner prepared by this process has good stability and ductility during jet forming.

Because the bonding between Cu and PTFE particles is closer after hot-pressing sintering, the densification of hot-pressing sintering liner is better than that of extrusion molding^3^. If the two kinds of liners collapse into the same jet, the hot-pressing sintering liner needs higher detonation pressure. Therefore, under the same charge condition, the jet length of the hot-pressing sintering liner is always smaller than that of the extrusion molding liner during the jet stretching process.

When the density is 4 g/cm^3^, due to the difficulty of preparing the liner by extrusion molding, only the jet length data formed by the molded sintering and hot-pressing sintering liners are available. It can be seen from the figure that the length of the two jets is not much different at 10 μs. At 20 μs, the jet forming is stable. At this time, the jet length of the 8# liner is 91 mm, and the jet length of the 11# liner is 90.5 mm. With the increase of time, the jet length formed by the molded sintering liner is gradually larger than that of the hot-pressing sintering liner. At 50 μs, the jet length of the 8# liner is 267.79 mm, and the jet length of the 11# liner is 262.24 mm.

### Pulse X-ray experiment results

According to the above parameter settings, the pulse X-ray experiment of the liner forming is carried out respectively. As shown in the following figure, the X-ray photos of the jet forming state of different liners at two trigger times and the corresponding simulation results are shown respectively. The setting of the theoretical trigger time is shown in Table 3, and the trigger time in the actual experiment is delayed due to the existence of the initiation error. The specific time is shown in the following figures.

**Figure 21:**
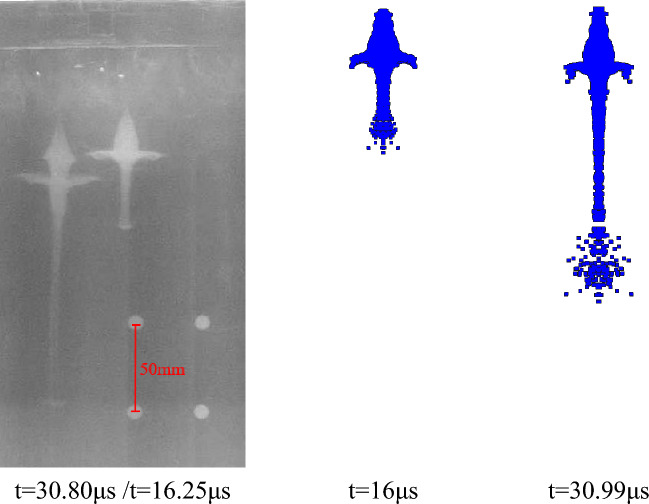
X-ray and simulation results of jet forming of 2#-PTFE/Cu liner (Extrusion molding, $\rho$ = 3 g/cm^3^).

Figure 21 shows the jet forming X-ray image of the extrusion molding liner with a density of 3.5 g/cm^3^ and the corresponding simulation results. It can be seen from the figure that the actual trigger time during detonation is slightly delayed than the theory. This error is due to the experiment environment. The X-ray morphology of the jet forming is similar to the simulation results. With the passage of time, the radial expansion at jet head gradually weakens, the jet forming is stable and the symmetry is good, which is consistent with the simulation analysis results.

**Figure 22:**
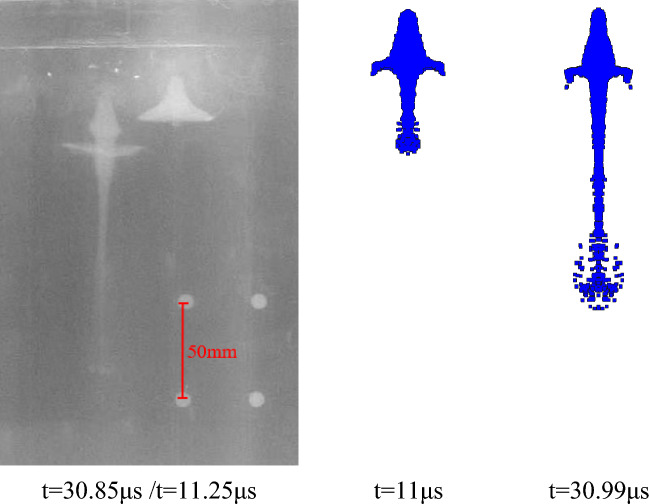
X-ray and simulation results of jet forming of 4#-PTFE/Cu liner (Molded sintering, $\rho$ = 3.5 g/cm^3^).

**Figure 23:**
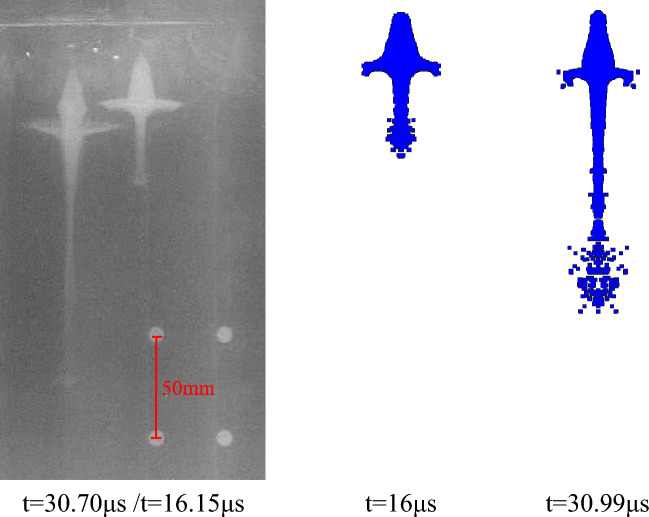
X-ray and simulation results of jet forming of 5#-PTFE/Cu liner (Molded sintering, $\rho$ = 4 g/cm^3^).

Figures 22 and 23 are the jet X-ray photos and simulation results of the molded sintering liner with a density of 3.5 g/cm^3^ and 4 g/cm^3^, respectively. It can be seen from the diagram that the symmetry of the jet forming is good and there is no fracture, indicating that the processing error of the liner prepared by molded sintering is small. At the same time, because the density of the jet head is less than the density of the slug, the imaging color of the jet head in the X-ray image gradually becomes lighter as time increases. In addition, compared with Figs. 22 and 23, it can be seen that with the increase of density, the head color of the jet gradually deepens. This is due to the increase of the proportion of Cu, which has exceeded half of the total mass of the liner, making the forming characteristics of the jet gradually close to the characteristics of the conventional metal jet.

**Figure 24:**
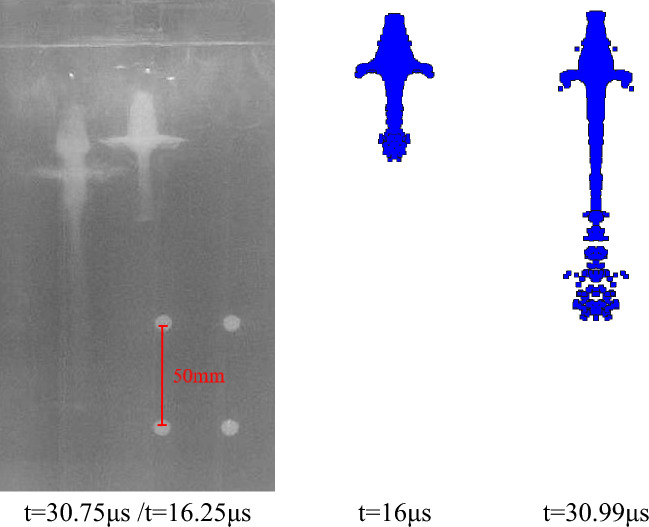
X-ray and simulation results of jet forming of 7#-PTFE/Cu liner (Hot-pressing sintering, $\rho$ = 3.5 g/cm^3^).

**Figure 25:**
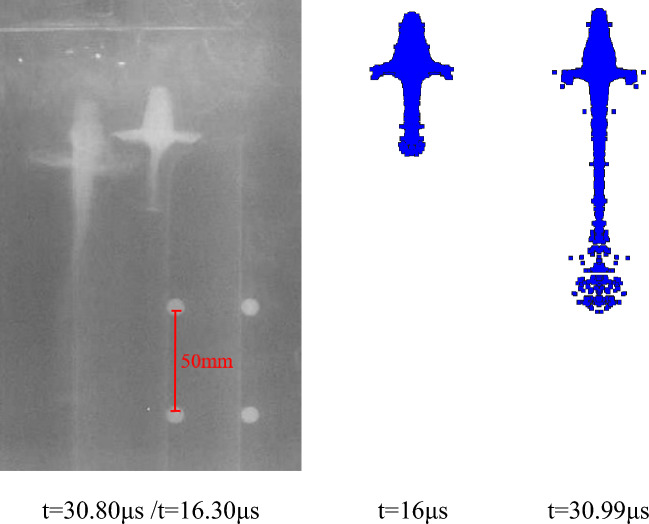
X-ray and simulation results of jet forming of 8#-PTFE/Cu liner (Hot-pressing sintering, $\rho$ = 4 g/cm^3^).

Figures 24 and 25 are the X-ray photos and simulation results of the jet formed by the hot-pressing sintering liner with a density of 3.5 g/cm^3^ and 4 g/cm^3^, respectively. The recorded time is the actual trigger time of the experiment. It can be seen from Fig. 24 that the X-ray photos of the jet morphology are close to the simulation results. However, there are some differences between the X-ray photos of Fig. 25 and the simulation results. The main reason is that the X-ray photos of the jet are offset at 30.8 μs, and the jet is asymmetric as a whole. There are two possibilities for this situation: First, the machining error caused by the processing of the liner makes it uneven in the axial or radial direction, resulting in uneven wall thickness of the liner, which makes the liner unevenly stressed when it is crushed by the detonation wave; the second is the error caused by the bonding during the experiment. The shellac paint is used to bond between the liner and the charge. Due to the existence of processing errors, it may not be closely bonded. In addition, it can be seen that the bonding of the experiment is carried out by tape. The coincidence of the detonator face and the grain and the parallelism of the shaped charge face and the stand-off barrel will bring inevitable errors, which makes the detonation wave and the liner appear asymmetric. These factors can affect the stable formation of the jet, resulting in fracture and asymmetry, thereby reducing the penetration power of the jet.

After comparing the jet forming shape, according to the fixed position of the steel ball, the amplification ratio of different shaped charge liners is calculated, and then the distance between the jet heads at two different times is read from the figure. The length between the actual jet heads is calculated by using the amplification ratio and the pixel distance, and the time difference is known. Therefore, the average velocity of the jet head through this distance is calculated. At the same time, according to the numerical simulation results, the simulation velocity of the jet head in this period is determined. The two data are compared to analyze the velocity error between the experiment and the theory, and the accuracy of the material model and the equation of state parameters of the shaped charge liner is verified in reverse.

It can be seen from Table 7 that the difference between the jet head velocity measured by the X-ray experiment of the extrusion molding liner and the simulation result is the smallest, which is 1.29%. The velocity errors of hot-pressing sintering and molded sintering liner are 1.43%, 2.08% and 1.62%, 2.96%, respectively. In addition to the unavoidable errors in the experiment, the material model of the liner and the parameter selection of the equation of state in the numerical simulation will also lead to the result. In addition, according to the data measured by X-ray, it can be seen that for the liners of the three preparation processes, the jet velocity of the molded liner is the largest, followed by hot-pressing sintering, and finally extrusion molding. With the increase of density, the change of the simulated velocity of the jet in the molded sintering and hot-pressing sintering is consistent with the change of the velocity measured by X-ray, which indicates that the machining error of the two processes is small, and the simulated parameters are also effective.

**Table 7:** Comparison between experimental results and simulation results of jet forming.

Liner type	X-ray calculation velocity (m/s)	Simulation velocity (m/s)	Velocity error (%)
Extrusion molding ($\rho$ = 3.5 g/cm^3^)	6397.9	6482	1.29
Molded sintering ($\rho$ = 3.5 g/cm^3^)	6534.7	6642	1.62
Molded sintering ($\rho$ = 4 g/cm^3^)	6191.8	6380	2.96
Hot-pressing sintering ($\rho$ = 3.5 g/cm^3^)	6413.8	6507	1.43
Hot-pressing sintering ($\rho$ = 4 g/cm^3^)	6106.9	6236	2.08

## Conclusions

In this paper, the jet forming of PTFE/Cu liners with different preparation processes was numerically simulated, and the verification experiment of pulse X-ray was carried out. The main results are as follows:

1. The simulation results of jet forming show that the PTFE/Cu jet head has obvious radial expansion effect, but the increase of density means the increase of Cu content, so the jet formed by PTFE/Cu liner can more and more reflect the characteristics of copper jet, and the radial expansion effect is weakened. With the increase of density, the jet velocity decreases. With the increase of Cu content in the liner, the forming dispersion of the jet is weakened, and the cohesiveness is enhanced. However, no matter how the preparation process is selected, because the densification of the molded sintering liner is higher than that of the other two processes, the velocity of the formed jet head is always the highest.
2. The results of pulse X-ray experiment show that the simulation results of jet morphology are close to the X-ray photos, which verifies that the numerical simulation results are effective. Compared with the results measured by the experiment, the velocity error of the jet velocity calculated by the simulation is controlled within 2.96%.
3. Combining the results of numerical simulation with the results of pulse X-ray experiment, it can be obtained that for the liners of the three preparation processes, the jet velocity from large to small is molded sintering liner, hot-pressing sintering liner and extrusion molding liner.

## Data Availability

All data generated or analyzed during this study are included in this published article.
